# Letrozole and ovarian hyperstimulation syndrome: Retrospective cross-sectional study

**DOI:** 10.18502/ijrm.v22i3.16165

**Published:** 2024-05-15

**Authors:** Elham Nikfarjam, Maryam Eftekhar, Hanieh Fatehi, Sahereh Arabian

**Affiliations:** ^1^Research and Clinical Center for Infertility, Yazd Reproductive Sciences Institute, Shahid Sadoughi University of Medical Sciences, Yazd, Iran.; ^2^Research Center for Abnormal Uterine Bleeding, Semnan University of Medical Sciences, Semnan, Iran.

**Keywords:** Letrozole, Polycystic ovary syndrome, Ovarian hyperstimulation syndrome, Assisted reproductive technologies.

## Abstract

**Background:**

Recently, letrozole has been used to prevent moderate to severe ovarian hyperstimulation syndrome (OHSS) in assisted reproductive technology cycles due to its estrogen-reducing and androgen-increasing effects on the ovaries, affecting granulosa cells, and reducing vascular endothelial growth factor production.

**Objective:**

This study aimed to investigate the impact of letrozole consumption in preventing OHSS in infertile women with polycystic ovarian syndrome undergoing in vitro fertilization.

**Materials and Methods:**

In this cross-sectional study, among 1743 medical records of infertile women who were scheduled for oocyte retrieval at Research and Clinical Center for Infertility, Yazd, Iran. Data of 343 women with polycystic ovarian syndrome diagnosis and at risk of OHSS was extracted from March 2022–2023. The stimulation was carried out using a flexible gonadotropin releasing hormone (GnRH) antagonist protocol. Women were divided into 2 groups based on whether they received letrozole or not. In the letrozole group, 2.5 mg letrozole twice daily was continued from the trigger day, while in the control group, women did not receive letrozole. The parameters of OHSS severity, hospitalization rates, and the need for albumin prescription were analyzed.

**Results:**

89 women in the letrozole and 254 women in the control group were examined. There was no statistically significant difference between groups in terms of age and body mass index; however, anti-Mullerian hormone was significantly higher than control group (7.53 ± 4.61 vs. 5.47 ± 3.63, p < 0.001). The parameters of OHSS severity, hospitalization rates, and the need for albumin prescription showed no significant differences between the groups.

**Conclusion:**

Recent study indicates that incorporating letrozole into the treatment of GnRH antagonists and cabergoline does not reduce the OHSS severity.

## 1. Introduction

Ovarian hyperstimulation syndrome (OHSS) is one of the significant side effects of controlled ovarian stimulation. The main factor causing OHSS is the use of human chorionic gonadotropin (HCG) hormone, which initiates early OHSS (1). The HCG induces final oocyte maturation before egg retrieval. The half-life of HCG (36 hr) is longer than that of luteinizing hormone (20 min), resulting in a more prolonged luteotropic effect on ovaries (2–4).

HCG is a key event in increasing the synthesis of inflammatory mediators and vasoactive substances. Among them, vascular endothelial growth factor plays an essential role in the development and exacerbation of OHSS by increasing vascular permeability (2, 4, 5).

Increased vascular permeability leads to extravasation of fluid from the vessels into the extravascular space (3 ${\;}^{\text{rd}}$ space), resulting in Ascites (1). Moderate to severe OHSS is observed in approximately 1–5% of in vitro fertilization cycles, with rates reaching up to 20% in high risk women (6). The risk factors for OHSS are divided into 2 groups: primary risk factors (woman-related) that include: young age (age ≤ 35), low body mass index, increased anti-Mullerian hormone ( > 3.36 ng/ml), polycystic ovarian syndrome (PCOS) and previous history of OHSS.

The secondary risk factors are related to ovarian response to controlled ovarian hyperstimulation (2, 3, 5). OHSS is classified into 2 types depending on the timing of symptom onset: 1) Early onset occurs within 3–7 days after administrating exogenous HCG to stimulate ovaries for triggering ovulation. 2) Late onset occurs after 10 days of administrating exogenous HCG, and it may take place if pregnancy happens (1, 6). Based on clinical manifestations and laboratory findings, OHSS is categorized into: mild, moderate, severe, and critical cases (3).

Various approaches have been employed to minimize the incidence of OHSS, including the application of a minimal dose and duration of gonadotropin to achieve optimal follicular growth implementing a step-up (increasing) protocol, applying a gonadotropin releasing hormone (GnRH)-antagonist protocol, triggering with GnRH-agonist in the GnRH-antagonist protocol, freezing all embryos as a policy, coasting and utilizing in vitro maturation (7–9).

Therefore to prevent OHSS, the best approach is identifying OHSS risk factors in the woman and then adjusting ovarian stimulation protocols accordingly and choosing the best protocol tailored to high-risk women (10, 11).

Previous studies have introduced various drugs such as calcium, glucocorticoids, hydroxy ethyl starch, albumin, cabergoline, and letrozole for preventing OHSS. However, there is a disagreement regarding the effectiveness of these drugs in preventing OHSS, and a conclusive result is not established (7–9, 12–14). Letrozole is the third generation, non-steroidal aromatase inhibitor binding reversibly to the aromatase enzyme; thereby inhibiting the conversion of androgen to estrogen and reducing blood estradiol levels by approximately 98%. The negative estrogen feedback on hypothalamus and pituitary is controlled by letrozole, leading to increased secretion of GnRH, subsequently stimulating follicle-stimulating hormone and luteinizing hormone release. This mechanism is utilized by letrozole to induce ovulation. Aromatase inhibitors has no effect on estrogen receptors in the endometrium and the brain, while keeping the central feedback mechanism intact (15–17).

Letrozole has recently gained attention for preventing moderate to severe OHSS in assisted reproductive technologies (ART) cycles, due to its estrogen-reducing and androgen-increasing effects, affecting granulosa cells and reducing vascular endothelial growth factor production (18).

This retrospective and cross-sectional study, aimed to examine the efficacy of adding letrozole from the trigger day in preventing OHSS in women with PCOS and infertility undergoing ART treatments, particularly those at risk of OHSS.

## 2. Materials and Methods

In this retrospective cross-sectional study, among 1743 medical records of infertile women who were scheduled for oocyte retrieval at the Research and Clinical Center for Infertility, Yazd, Iran, data of 343 women (with PCOS diagnosis and risk of OHSS) was extracted from March 2022–2023.

### Inclusion and exclusion criteria

Infertile women with PCOS, according to Rotterdam criteria (19) undergoing ART cycles, aged ≤ 40 yr, and having estradiol level ≥ 3000 pg/ml and or ≥ 15 dominant follicles, on trigger day. Women with severe endometriosis and those who utilized donated oocytes were excluded from the study.

### Sample size

The names of 346 infertile women diagnosed with polycystic ovary syndrome undergoing in vitro fertilization treatment and at risk of ovarian hyperstimulation syndrome were extracted from the computer (electronic medical record system). File for 343 individuals were examined, but 3 files were missing and excluded from the study.

### Stimulation protocol

**The stimulation was carried out using a flexible GnRH antagonist protocol. Recombinant-follicle stimulating hormone or human menopausal gonadotropin at a daily dosage of 150–225 units was initiated from cycle day 2. Individuals monitoring and assessment were conducted using transvaginal ultrasound from the 6 ${\;}^{\text{th}}$ day of the menstrual cycle, when one follicle of 14 mm was seen, a daily subcutaneous injection of 0.25 mg of GnRH antagonist was administrated until the scheduled trigger time. Based on ultrasound criteria indicating the presence of 2–3 follicles with a dimension of 17 mm, a dual trigger was administered using 1500 units of HCG and GnRH agonist. Following the trigger, 34–36 hr later, ovarian puncture under general anesthesia and guided transvaginal ultrasound were performed. All embryos were cryopreserved on day 3 for transfer in subsequent cycles. Women were then monitored 2 days to 1 wk after ovarian puncture. Women were divided into 2 groups based on whether they received Letrozole or not. In the Letrozole group, a dosage of 2.5 mg twice daily was continued from the trigger day for 5 consecutive days, while in the control group, women did not receive letrozole. In both groups, after an ovarian puncture, a daily subcutaneous injection of 0.25 mg of GnRH antagonist for 3 consecutive days and vaginal administration of 0.5 mg cabergoline twice daily for 5 days were prescribed.**

### Classification of OHSS according to the severity of symptoms

1. Mild OHSS- bloating, mild abdominal distension or pain, the presence of fluid in the Douglas pouch (cul-de-sac), enlarged ovaries, mild nausea ± vomiting, and mild dyspnea.

2. Moderate OHSS- moderate lower abdominal pain, nausea ± vomiting, the presence of fluid around the uterus (ultrasonographic evidence of ascites), hemoconcentration ( > 45%), elevated white blood cells ( > 15000).

3. Severe OHSS- clinical evidence of Ascites with or without hydrothorax, oliguria (urine output < 30 cc per hour) or anuria, intractable nausea or vomiting, severe dyspnea, severe hemoconcentration (hematocrit > 55%), white blood cells > 25,000, creatinine > 1.6, sodium < 135 meq/lit potassium > 5 meq/lit, elevated liver enzymes and the presence of fluid between bowel loops.

4. Critical OHSS- rapid weight gain ( > 1 kg in 24 hr), low blood or central vein pressure, pleural effusion, severe abdominal pain, syncope, venous thrombo emboli, acute renal failure, adult respiratory distress syndrome, sepsis, massive hydrothorax, arterial thrombosis, arrhythmia.

### Ethical considerations

This study was approved by the Ethics Committee of the Research and Clinical Center for Infertility, Shahid Sadoughi University of Medical Sciences, Yazd, Iran (Code: IR.SSU.RSI.REC.1402.003).

### Statistical analysis

Statistical data analysis was done using SPSS version 26 software for windows (SPSS Inc, IL, USA). The Mann-Whitney test was employed to compare differences between continuous variables exhibiting a normal distribution. The Chi-square test and Fisher's exact test were employed for analyzing categorical variables. Data were displayed as mean ± SD and number (%) for continuous and categorical variables. The significant level in this study was denoted by p < 0.05.

## 3. Results

Out of 343 infertile PCOS women with risk of OHSS, 89 women in the letrozole group and 254 in the control group were included in this study. Women baseline characteristics are reported in table I. There was no statistically significant difference in terms of age and BMI. However, the letrozole group exhibited a significantly higher anti-Mullerian hormone level compared to the control group (7.53 ± 4.61 vs. 5.47 ± 3.63, p < 0.001) (Table I).

Table II shows the characteristics of ovulation stimulation cycles. There were no significant differences in terms of infertility duration, type of infertility, 2 pro nucleus count, and embryo count. However, a significant difference was observed in mature oocyte count between the letrozole and the control group (18.36 ± 8.46 vs. 14.88 ± 6.43, p < 0.001).

The severity parameters of OHSS, hospitalization rates, and the need for albumin prescription were reported in table III, showing no significant differences between groups. Considering that the 2 groups were not homogeneous in terms of ovarian stimulation cycle characteristics, cases were divided into 2 subgroups based on the number of obtained oocytes, less and more than 20. The parameters of OHSS severity in these groups were separately reanalyzed by a statistical expert, and the results showed no statistically significant difference in OHSS severity between cases with fewer or more than 20 oocytes in both groups (Table IV).

**Table 1:** Basic characteristics of study groups


**Variable**	**Letrozole group**	**Control group**	**P-value**
**Age (yr)**	29.71 ± 5.06 (29.50, 7.25)	30.20 ± 5.24 (30.00, 7.00)	0.54
**BMI (kg/m^2^)**	26.50 ± 4.88 (25.65, 6.40)	27.86 ± 18.26 (26.40, 6.25)	0.65
**AMH (ng/ml)**	7.53 ± 4.61 (6.80, 5.58)	5.47 ± 3.63 (4.40, 3.70)	< 0.001
Data presented as Mean ± SD (MD, IQR), Mann-Whitney test. BMI: Body mass index, AMH: Anti-Mullerian hormone			

**Table 2:** Cycle characteristics in study groups


**Variable**		**Letrozole group**	**Control group**	**P-value**
Infertility duration (yr)*		6.46 ± 4.24 (5.00, 6.00)	6.93 ± 4.02 (6.00, 6.00)	0.24
Cycle duration (days)*		11.74 ± 1.78 (12.00, 2.00)	11.06 ± 1.66 (11.00, 2.00)	0.006
Numbers of oocytes*		24.01 ± 8.74 (22.00, 10.00)	20.43 ± 6.02 (19.00, 7.00)	< 0.001
Numbers of mature oocytes*		18.36 ± 8.46 (16.50, 9.25)	14.88 ± 6.43 (14.00, 6.00)	< 0.001
Numbers of 2PN*		11.91 ± 8.44 (11.00, 10.00)	9.48 ± 6.43 (8.00, 7.00)	0.07
Numbers of embryos*		10.00 ± 7.06 (8.00, 7.25)	8.21 ± 5.91 (7.00, 7.00)	0.09
Estradiol levels (pg/ml)*		6568.95 ± 4181.69 (5646.00, 5172.00)	4546.73 ± 2817.25 (3822.00, 4404.50)	< 0.001
Type of infertility**				
	**Primary**	80 (89.9)	213 (83.9)	
	**Secondary**	9 (10.1)	41 (16.1)	0.17
*Data presented as Mean ± SD (MD, IQR), Mann Whitney test. **Data presented as n (%), Mann Whitney test, 2PN: 2 pro nucleus				

**Table 3:** Parameters according to severity of OHSS


**Variable**		**Letrozole group**	**Control group**	**P-value**
**Incidence rate of OHSS**				
	**Mild**	0 (0.0)	5 (2)	
	**Moderate**	77 (86.5)	219 (86.2)	
	**Severe**	12 (13.5)	30 (11.8)	0.39*
**Hospitalization**		12 (13.5)	31 (12.2)		0.754**
**Albumin administration**		12 (13.5)	28 (11)		0.534**
Data presented as n (%). *Fisher exact test, **Chi-square test, OHSS: Ovarian hyperstimulation syndrome				

**Table 4:** Parameters according to the severity of OHSS in subgroup numbers of oocytes


**Variable**		** ≤ 20**			** > 20**		
		**Letrozole group**	**Control group**	P-value*	**Letrozole group**	**Control group**	P-value**
**Incidence rate of OHSS**							
	**Mild**	0 (0.0)	5 (3)	0 (0.0)	0 (0.0)	
	**Moderate**	32 (91.4)	153 (93.3)	45 (83.3)	66 (73.3)	
	**Severe**	3 (8.6)	6 (3.7)	0.270	9 (16.7)	24 (26.7)	0.167
**Hospitalization**		3 (8.6)	6 (3.7)		0.197	9 (16.7)		25 (27.8)	0.129
**Albumin administration**		3 (8.6)	6 (3.7)		0.197	9 (16.7)		22 (24.4)	0.272
Data presented as n (%). *Fisher's exact test, **Chi-square test. OHSS: Ovarian hyperstimulation syndrome							

## 4. Discussion

Our study revealed no significant differences in OHSS severity parameters between the group receiving letrozole and the control group. Given the retrospective and cross-sectional nature of our study and the variable characteristics of participants ovarian stimulation cycles, we categorized women, based on the number of retrieved eggs, into 2 subgroups with less than or more than 20 eggs. Despite a separate statistical analysis for each subgroup, no significant differences were found in OHSS severity parameters between the 2 groups, regardless of whether the number of oocytes was less or more than 20.

The symptoms of OHSS are relieved by initiating luteolysis, and drug interventions are applied for both prevention and treatment following this approach (6).

In 2023 an RCT containing infertile women with PCOS undergoing ART cycles and at risk of OHSS, the effectiveness of combined treatment with letrozole, mifepristone, and GnRH antagonist was compared to a group using only mifepristone for OHSS prevention. Both groups used the mentioned drugs post trigger, and the combined therapy significantly reduced OHSS severity compared to the control group (17).

In an RCT in 2021, it was found that letrozole and GnRH antagonists during the luteal phase prevent OHSS severity in ART cycles (3). In another RCT women undergoing ART received letrozole or cabergoline for 1 wk post puncture; Letrozole significantly reduced estradiol levels on the 7 ${\;}^{\text{th}}$ day post puncture compared to the cabergoline group, but did not demonstrate superior effectiveness in preventing OHSS (10).

In a retrospective study similar to ours, PCOS women undergoing ART cycles were categorized into 2 groups. The group receiving a combination of letrozole, cabergoline, and GnRH antagonist for 5 consecutive days from the day of puncture demonstrated a statistically significant and meaningful effectiveness in preventing OHSS compared to the control group using GnRH antagonist protocol and agonist trigger. This was in contrast with the findings in our retrospective analysis (20).

In a study involving infertile women with PCOS undergoing ART with a long follicular phase GnRH agonist protocol, those with estradiol levels ≥ 4000 pg/ml were split into 2 groups. One group took 2.5 mg of letrozole daily, and it discontinued before triggering with HCG, while the control group did not receive letrozole. Both groups showed no significant statistical difference in preventing OHSS. Administrating letrozole in the follicular phase did not effectively reduce OHSS incidence (18). However, an RCT demonstrated that using letrozole for 5 days in the early follicular phase of A GnRH antagonist protocol led to a significant reduction of OHSS (21).

In our study, the addition of letrozole did not enhance outcomes related to OHSS parameters. However, given prior researches indicating that letrozole reduces estradiol levels and expedites luteolysis, substituting letrozole for GnRH antagonist in OHSS prevention not only lowers treatment costs but also eliminates the need for frequent injections, making it a more patient friendly option.

## 5. Conclusion

A recent study indicates that incorporating letrozole into the treatment of GnRH antagonists and cabergoline does not lead to a reduction in the severity of OHSS. Conducting RCTS is essential for comparing letrozole with other therapeutic approaches to prevent the onset of OHSS.

##  Data availability

The data that support the findings of this study are available upon reasonable request from the corresponding author (M.E.).

##  Author contributions

Elham Nikfarjam: drafting of the manuscript, concept, and design, Maryam Eftekhar: had full access to all the data in the study and took responsibility for concept and design, the integrity of the data and the accuracy of the data analysis. Hanieh Fatehi and Sahereh Arabian: acquisition, analysis, or interpretation of data. All authors: drafting of the manuscript, critical revision of the manuscript for important intellectual content.

##  Conflict of Interest 

The authors declare that there is no conflict of interest.
